# Supplementary material for: Quantitative nylon monomerization by the combination of chemical pretreatment and enzymatic hydrolysis using nylon hydrolases
Source: PLoS One. 2025 Feb 10;20(2):e0318641. doi: 10.1371/journal.pone.0318641 (PMC11809917; doi:10.1371/journal.pone.0318641)
Supplement: S1 File — (PDF) [file pone.0318641.s001.pdf]

Supporting information

Quantitative nylon monomerization by the combination of chemical pretreatment and enzymatic hydrolysis using nylon hydrolases

Yuki Shiraishi[1], Dai-ichiro Kato[1]*, Kaito Miyazaki[1], Maina Yonemura[1], Yoko Furuno[1], Risa Yokoyama[1], Yukiko Yokogawa[1], Sho Nonaka[2], Yoshiro Kaneko[2], Keigo Ebata[3], Yuichiro Himeda[4], Seiji Negoro[5]

[1] Department of Science, Graduate School of Science and Engineering, Kagoshima University, Korimoto, Kagoshima, Japan
[2] Department of Engineering, Graduate School of Science and Engineering, Kagoshima University, Korimoto, Kagoshima, Japan
[3] Faculty of Fisheries, Kagoshima University, Shimoarata, Kagoshima, Japan
[4] Global Zero Emission Research Center, National Institute of Advanced Industrial Science and Technology, Tsukuba, Ibaraki, Japan
[5] Department of Applied Chemistry, Graduate School of Engineering, University of Hyogo, Himeji, Hyogo, Japan

*Corresponding author: kato@sci.kagoshima-u.ac.jp (DK)

Table of Contents

1. Enzyme preparation 2
2. Homogeneous dispersion of nylons in aqueous solution 3
3. Soluble oligomerization of polymeric nylons by acid hydrolysis 4
4. Combination of homogeneous dispersion and formic acid caused soluble oligomerization 6
5. Average molecular weight of nylon after chemical pretreatment 7
6. Thermogravimetric analysis (TGA) of acid treated nylons 8
7. Verification of applicability toward used fishing net 9

1. Enzyme preparation

To test for the ability to hydrolyze nylon-6 and nylon-6,6, the N-terminal His-tag fused proteins were prepared as follows:

The NylB-DNY gene was amplified from pHY3DN1 plasmid by PCR with forward (CGGAGAGCATGCTTGAACGCACGTTCCACCGGCCAGC) and reverse (GGAGCGAAAGCTTCTACACTGCGTCGAGCGCGCGG) primers.[Sr1-Sr3] These primers were designed to contain artificially introduced Sph I and Hid III sites (shown in italics) in the 5′ and 3′ ends of the gene. The initial codon is underlined. The amplified fragment was digested with Sph I and Hid III, and then purified and inserted into a pQE-80L expression vector (Qiagen, Germany). The resultant plasmid, pHY3ADN1H, was used to express the recombinant protein. Escherichia coli BL21(DE3) cells were transformed with this constructed vector, cultivated, and used for recombinant protein expression by a general procedure.

The NylC-GYAQ gene was amplified from pSKFC4-GYAQ plasmid by PCR with forward (TCATCATATCGAAGGTAGGatgaatacgacaccggtccacgcac) and reverse (CCTATCTAGACTGCAGGTCGACctacttgccggcctcgaggacggc) primers.[16] The amplified fragment was ligated into the expression vector pCold I (Takara Bio, Kusatsu, Japan) using the SLiCE method.[Sr4] The resultant plasmid, pColdI-GYAQ, was used to express the recombinant protein. Escherichia coli BL21(DE3) cells were transformed with this expression vector, cultivated, and used for recombinant protein expression by a cold shock procedure.

After production of the recombinant enzymes, the cells were harvested and disrupted by sonication (20 kHz, 30 s × 10 times) in 50 mM potassium phosphate buffer (pH 7.0) containing 300 mM NaCl. After centrifugation (14,500 × g for 10 min, 4°C), the target protein was purified from the supernatant using TALON Metal Affinity Resin (Clontech, Mountain View, CA, USA) according to the manufacturer’s instructions. Active fractions were combined and dialysed overnight in buffer A (20 mM potassium phosphate buffer (pH 7.3) containing 10% glycerol). In case of NylC-GYAQ, additonal 24 hr incubation at 37 °C was performed for complete enzyme activation. Protein concentrations were measured using Bio-Rad Protein Assay Dye Reagent (Bio-Rad, Hercules, CA, USA) with bovine serum albumin as a standard. The purified protein was stored at −30°C until use.

2. Homogeneous dispersion of nylons in aqueous solution

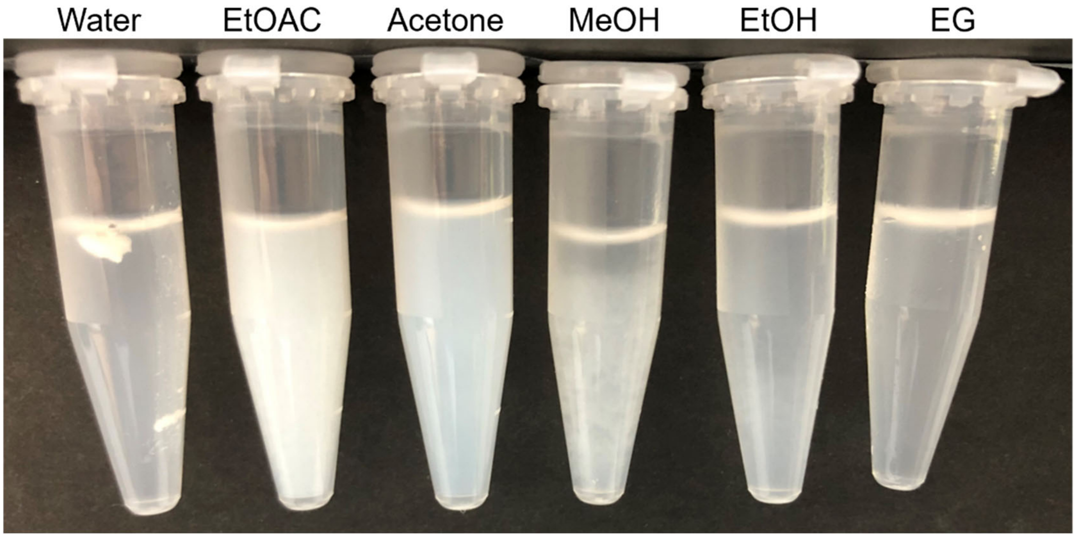

S1 Fig. Differences in the degree to which the homogeneous dispersion of nylon-6 dropped in six poor solvents. From left to right: water, EtOAc, acetone, MeOH, EtOH, and EG.

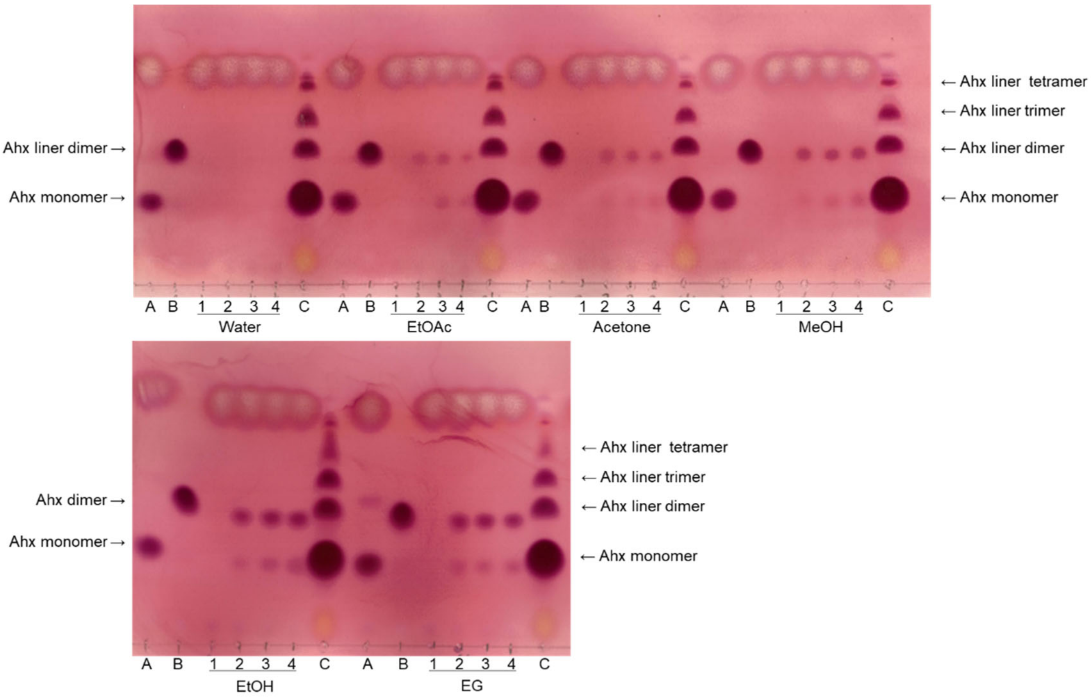

S2 Fig. TLC analysis of NylC-GYAQ treated reaction products toward homogeneous dispersed nylon-6 in water, EtOAc, Acetone, MeOH, EtOH and EG. A: Ahx (monomer),

B: Ald (Ahx linear dimer), C: Ahx linear oligomer, 1: 0 hr, 2: 24 hr, 3: 48 hr, 4: 72 hr.

3. Soluble oligomerization of polymeric nylons by acid hydrolysis

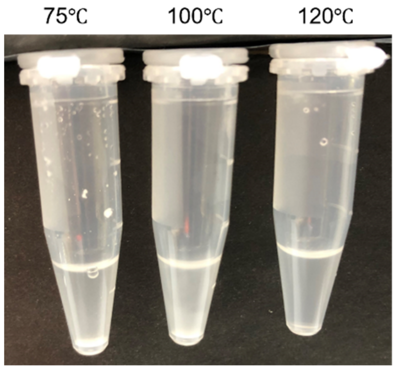

S3 Fig. Differences in the dispersion level of resuspended nylon-6 in buffer A. From left to right: 75 ℃, 100℃, 120℃.

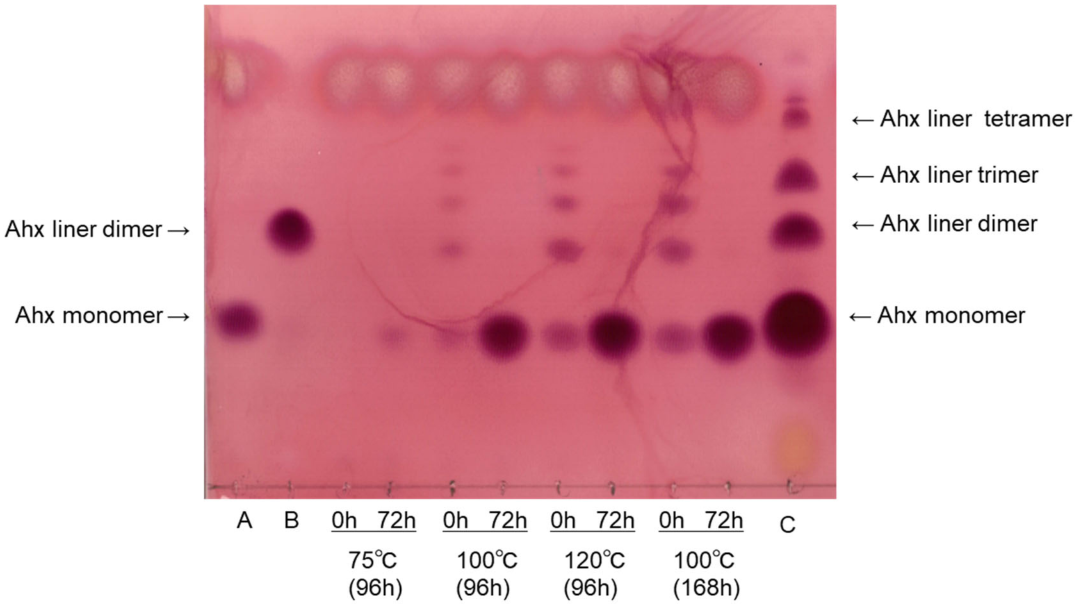

S4 Fig. TLC analysis of NylC-GYAQ and NylB-DNY treated reaction products toward soluble oligomerized nylon-6 at various temperatures and reaction times. From left to right: 75°C/96 hr, 100°C/96 hr, 120°C/96 hr, and 100°C/168 hr. A: Ahx (monomer), B: Ald

(Ahx linear dimer), C: Ahx linear oligomer.

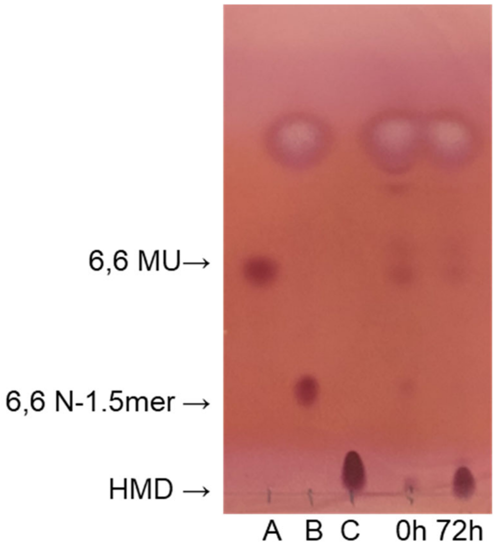

S5 Fig. TLC analysis of NylC-GYAQ and NylB-DNY treated reaction products toward soluble oligomerized nylon-6,6 at 100°C/168 hr. A: 6,6-MU, B: 6,6N-1.5mer, C: HMD.

4. Combination of homogeneous dispersion and formic acid caused soluble oligomerization

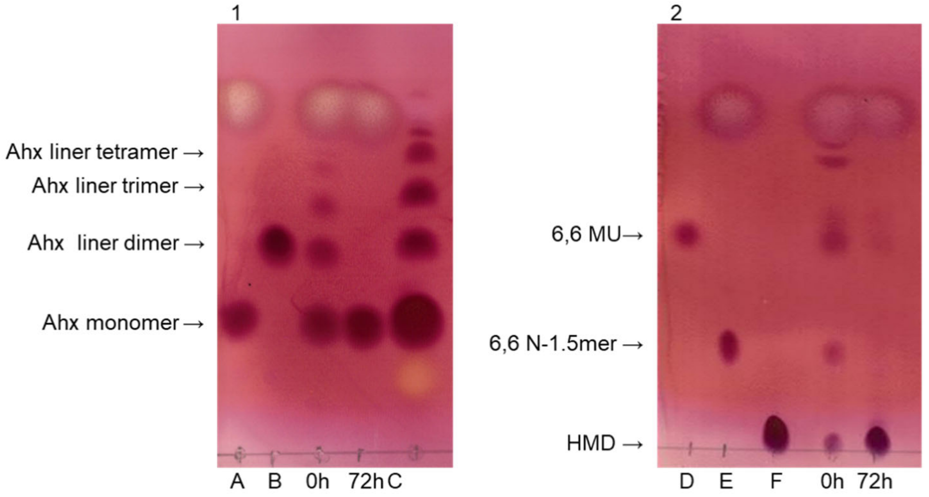

S6 Fig. TLC analysis of NylC-GYAQ and NylB-DNY treated reaction products toward combination chemical treatment nylon-6 and nylon-6,6 (homogeneous dispersion in EtOH and soluble oligomerization at 100°C/168hr). 1: nylon-6, 2: nylon-6,6. A: Ahx (monomer), B: Ald (Ahx linear dimer), C: Ahx linear oligomer, D: 6,6-MU, E: 6,6N-1.5mer, F: HMD.

5. Average molecular weight of nylon after chemical pretreatment

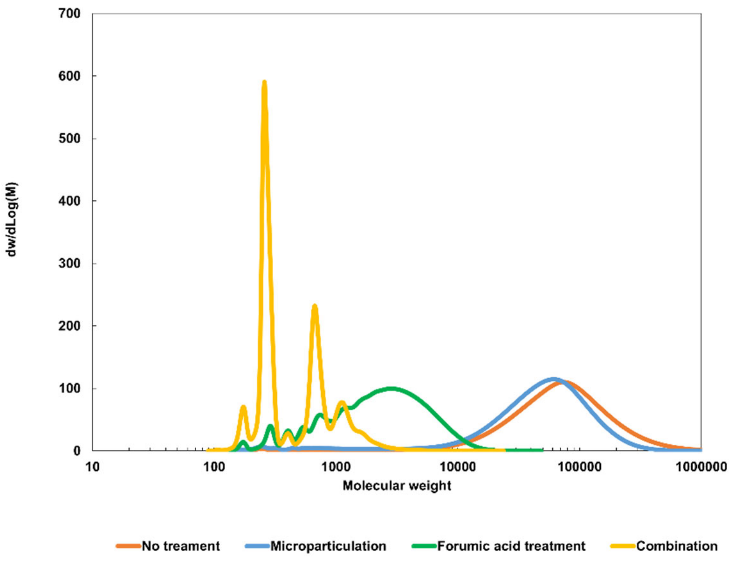

S7 Fig. Average molecular weight and its abundance distribution change of nylon-6 by chemical pretreatment.

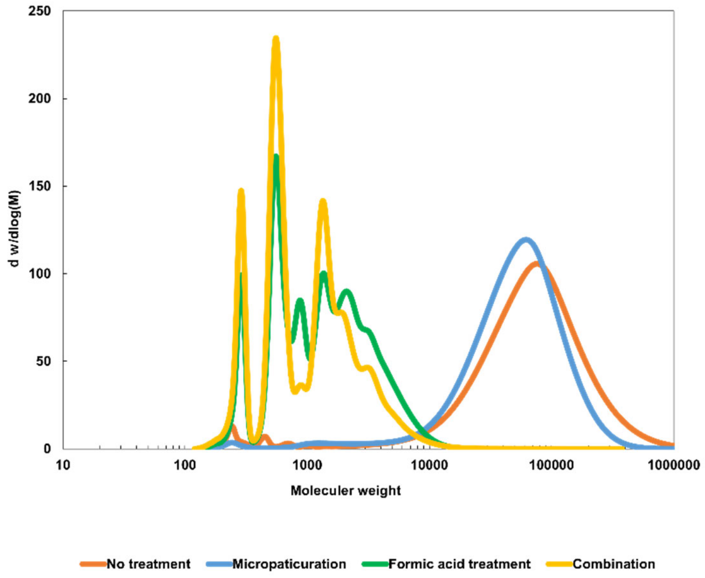

S8 Fig. Average molecular weight and its abundance distribution change of nylon-6,6 by chemical pretreatment.

6. Thermogravimetric analysis (TGA) of acid treated nylons

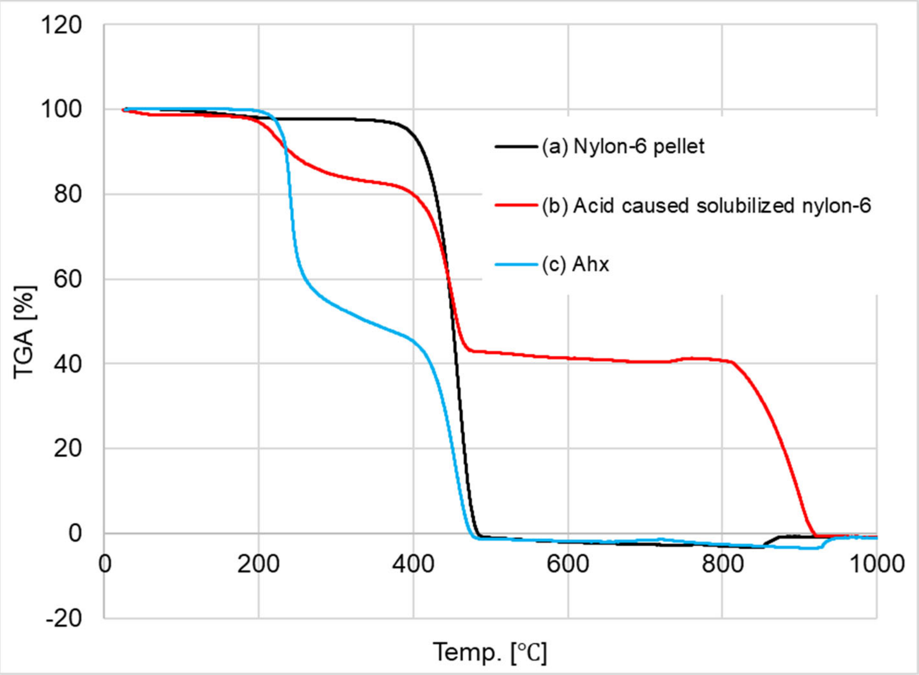

S9 Fig. TGA of nylon-6 pellet (a), 18% hydrochloric acid caused solubilized sample (b), and Ahx (c).

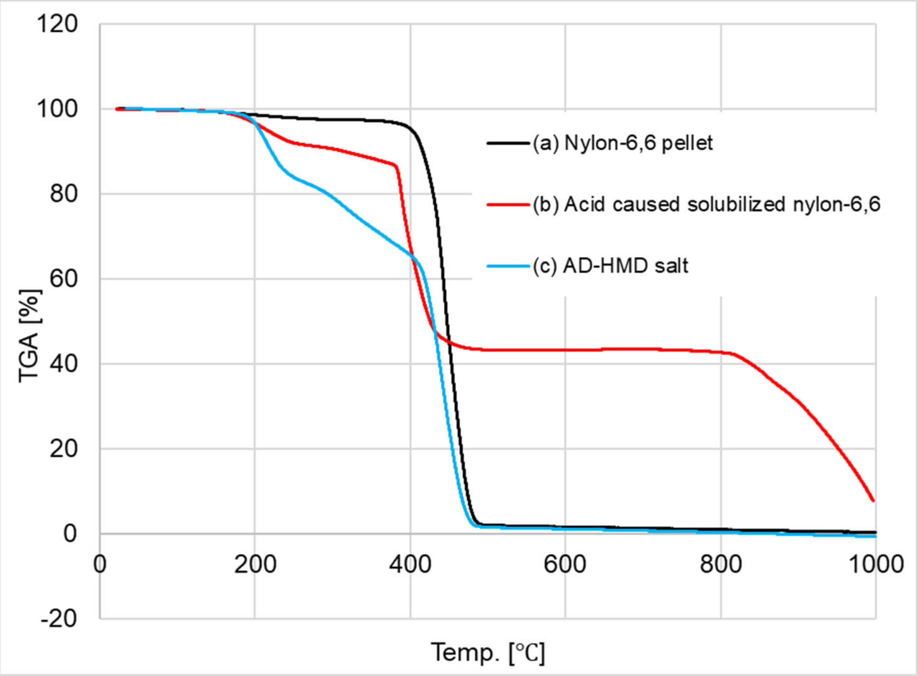

S10 Fig. TGA of nylon-6,6 pellet (a), 18% hydrochloric acid caused solubilized sample (b), and AD-HMD salt (c).

7. Verification of applicability toward used fishing net

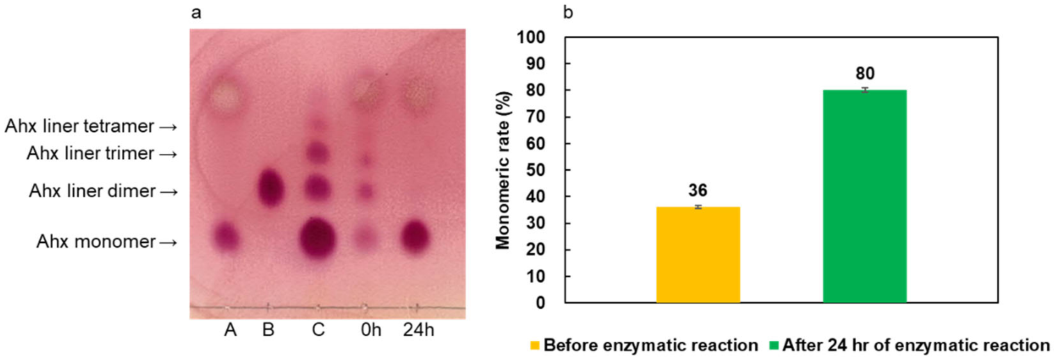

S11 Fig. TLC analysis (a) and monomeric rate (b) of 18% hydrochloric acid caused soluble oligomerization of fishing net before and after Nyl series catalyzed enzymatic hydrolysis. The experiments were conducted in triplicate (N=3). A: Ahx (monomer), B: Ald (Ahx linear dimer), C: Ahx linear oligomer.

References in supporting section

Sr1. Negoro S, Ohki T, Shibata N, Mizuno N, Wakitani Y, Tsurukame J, et al. X-ray Crystallographic Analysis of 6-Aminohexanoate-Dimer Hydrolase. J. Biol. Chem. 2005;280: 39644–39652.

Sr2. Negoro S, Ohki T, Shibata N, Sasa K, Hayashi H, Nakano H, et al. Nylon-oligomer Degrading Enzyme/Substrate Complex: Catalytic Mechanism of 6-Aminohexanoate-dimer Hydrolase. J. Biol. Chem. 2007;370: 142–156.

Sr3. Ohki T, Shibata N, Higuchi Y, Kawashima Y, Takeo M, Kato D, et al. Two alternative modes for optimizing nylon‐6 byproduct hydrolytic activity from a carboxylesterase with a β‐lactamase fold: X‐ray crystallographic analysis of directly evolved 6‐aminohexanoate‐dimer hydrolase. Protein Sci. 2009;18: 1662‐1673.

Sr4. Zhang Y, Werling U, Edelmann W. Seamless Ligation Cloning Extract (SLiCE) Cloning Method. Methods Mol. Biol. 2015;1116: 235–244.
