# Supplementary material for: Quantitative nylon monomerization by the combination of chemical pretreatment and enzymatic hydrolysis using nylon hydrolases
Source: PLoS One. 2025 Feb 10;20(2):e0318641. doi: 10.1371/journal.pone.0318641 (PMC11809917; doi:10.1371/journal.pone.0318641)
Supplement: Attachment — Submitted filename: Response to Reviewers.pdf [file pone.0318641.s002.pdf]

To the editor and reviewers,

First of all, we would like to express our sincere gratitude to all four reviewers for their insightful and constructive comments. Now we carefully revised our manuscript according to the reviewers' suggestions. Thanks to reviewers' feedback, we believe we have been able to provide readers with more accurate and detailed information than in the original submission.

To address the revisions, it was necessary to perform additional 1H-NMR analysis. As a result, we would like to propose adding the researcher, Yukiko Yokogawa, who conducted this experiment as a co-author. We have obtained consent from the other co-authors, and we kindly ask for reviewers' consideration on this matter.

Below, we provide our responses to each comment, color-coded as follows: Reviewer comments are provided in black font. Our responses are shown in blue font. Changes to the manuscript are shown in red font.

Reviewer #1: The manuscript provides new scientific insights into the degradation of polyamides (nylon) with enzymes (nylonases). To this date, the recycling and degradation of these polymer remains a major problem. Although there are a few somewhat effective chemical methods to degrade these polymers, as the authors mention correctly: "the degradation efficiency of enzymes for bulk polymeric nylon is limited to only a few percent at best." In that aspect, the results provided in this manuscript are a giant leap forward, since they were able to completely depolymerize nylon 6 and nylon 66. However, the following comments/questions should be addressed before I would recommend this article for publication:

1. In the introduction, the authors state "Nylon-6 and nylon-6,6 are composed of simple monomer units such as 6-aminohexanoate (Ahx) for nylon-6 and hexamethylenediamine and adipic acid for nylon-6,6,respectively." Although I understand that the authors want to introduce the "Ahx" abbreviation, this sentence gives the impression that nylon-6 is made from Ahx while in reality it is caprolactam. I would suggest to introduce a little nuance.

We thank the reviewer for their comment. We inserted and changed around sentences as following in introduction (Line 37-).

These monomer units can be re-polymerized into polymers either in their original form or after lactamization. As a result, chemical recycling technologies that break down polymeric nylons into these monomer units hold significant potential for advancing circular economic systems.[3]

2. In the sentence "NylB is an enzyme that hydrolyzes Ahx oligomers in an exo-type mode, and NylC

degrades Ahx cyclic and -linear oligomers with a degree of polymerization greater than three in an endo-type mode" the authors should clarify what they mean with "exo" and "endo"-mode.

We thank the reviewer for their suggestion. We changed to the following sentence in introduction (Line 50-).

NylB is an exo-type mode enzyme that cleaves amide bonds at the N-terminus of Ahx-linear oligomers to sequentially produce Ahx monomers.[14,15] NylC degrades Ahx-cyclic and -linear oligomers with a degree of polymerization greater than three in an endo-type mode and cleaves the internal amide bonds in oligomers to produce dimeric or higher linear oligomers.[16,17]

3. Nylon-6 and Nylon-6,6 are known to dissolve in formic acid. A) The authors should provide a blank reaction with nylon dissolved in formic acid at room temperature and treat it as if it was hydrolysed at 75-120°C (section 3.1). B) In section 2, Homogeneous dispersion of nylon: is there a reason why polyamides were dissolved in TFE and not formic acid? Formic acid is significantly more environmentally friendly. The authors should at least try to obtain homogeneous dispersions of nylon based on dissolving it in formic acid and adding it to antisolvent. Are they as easily broken down as in the TFE case?

We sincerely thank the reviewer for their valuable comments and insights. The possibility of using formic acid for a homogeneous dispersion process had not occurred to us, as we had primarily considered its role in hydrolyzing the amide bonds in nylon polymers. We are grateful to the reviewer for bringing this interesting perspective to our attention.

As Reviewer 2 pointed out, formic acid-based fine dispersion has already been reported in recent literature. We have now included this information and the relevant citation in the main text (Line 152-).

Recently, a technique for the fine dispersions in aqueous solution of nylon-6 using formic acid has been developed, showing improved sensitivity to enzymatic hydrolysis.[24] Since formic acid has a concern to cleave amide bonds in polymeric nylon and reduce its molecular weight, we explored the use of TFE, another nylon-dissolving and low concern for cleaving amide bonds solvent, for dispersions in aqueous solution.

4. In section 3.2, the authors refer to the homogeneous Ir-catalyst as a formic acid decarboxylation catalyst. In my opinion it would be significantly more clear for the reader to refer to it as a dehydrogenation catalyst or formic acid decomposition catalyst, and clearly state that it breaks formic acid down in CO2 and H2.

We thank the reviewer for their suggestion. We added the following sentence in section 3.2 (Line 122).

This catalyst breaks formic acid down in CO_2 and H_2.

5. The authors use the term "poor solvent" (e.g. After 72hr enzymatic hydrolysis, the monomeric rates

increased depending on the poor solvent used.) I suggest to replace this term with the more correct "antisolvent".
We thank the reviewer for pointing out this word selection. We changed 5 positions in the main body.

6. In figure 2, it is revealed that Nylon66 did degrade more extensively in comparison with nylon 6 in their initial runs even though the particle size was bigger. Do the authors have an explanation why?
We thank the reviewer for their comments. We believe that the enzyme sensitivity of dispersed nylon in EG shows little difference between the two types, with degradation rates of 11% for nylon-6 and 14% for nylon-6,6. However, it is not yet clear whether this difference in degradation rates correlates with differences in the size of the nylon particles. One of our hypotheses is that the percentage of amorphous nylon on the particle surface may significantly affect the degradability by Nyl series enzymes, but we have not yet thought of a way to resolve this hypothesis.

7. How resistant are the enzymes to formic acid? Are they inhibited by it? Ideally one or two reference reaction are performed with deliberate addition of formic acid. Additionally, polyamide waste is rarely "pure" polyamide. It can contain plasticizers, other plastics, pigments or fillers (e.g. carbon black). Additional experiments to evaluate the effect of these compounds would greatly improve the quality and impact of their research.
We thank the reviewer for their comments. The Nyl series enzymes become inactivated in acidic conditions below pH 5. Therefore, in our enzymatic reactions, the system must be neutralized with a base, or as described in this article, the enzyme reaction should be initiated after removing formic acid via lyophilization.
The potential for inhibition by additives is another intriguing aspect. Given the wide range of nylon composite materials available, we are actively investigating this topic, including identifying which products to target for further study. We would like to publish a follow-up paper on this topic in the future. The primary aim of this paper, however, is to establish the chemo-enzymatic reaction conditions for nylon monomerization. Therefore, we hope you will agree that our study of actual products in this paper is limited to fishing nets only.

8. Correction of grammatical error: "After enzymatic hydrolysis with Nyl series enzymes, it was clear that this net was made by nylon-6 and the monomerization rate was 80%" -> made of nylon-6
We thank the reviewer for pointing out a grammatical error. Our mistake was corrected (Line 281).

9. Although it is unlikely that the authors made other products than the polyamide monomers, what this paper still lacks is some actual molecular proof that they indeed made some Ahx and hexamethylene diamine (the colorimetric method + TLC only proves that something resembling these

compounds reacted with the dye). The authors should make an effort to record a mass spectrum or provide an 1H-NMR of the molecules they made. Have the authors any idea how to isolate their products? Effective product isolation is an extremely important factor, especially since the authors believe that “this method holds great potential for realizing full-scale chemical recycling of nylon products in the market.”

We thank the reviewer for their comments. We have employed qualitative analysis via TLC and quantitative analysis using the TNBS method in our previous papers. We now, however, agree with your suggestion that it is important to confirm whether the product is indeed the desired Ahx, adipic acid, or hexamethylenediamine. Thus first, we showed the LC-MS analysis to confirm the formation of nylon oligomers after hydrochloric acid treatment, and demonstrate that they are broken down into monomeric components through the Nyl series treatment (S9-S12 Fig and added the sentences in main body at Line267-). Second, we used 1H-NMR analysis to confirm that the final product matches the authentic spectrum of Ahx for nylon-6, and hexamethylenediamine and adipic acid for nylon-66 (S13, 14 Fig and added the sentences in main body at Line270-).

For isolating monomers from aqueous solutions, various methods are also known and investigated including the use of ion-exchange resins and solvent precipitation, and we believe that these methods can be also acceptable for our system.

Next, the molecular species present in the reaction mixture were tracked using LC-MS analysis (S9-S12 Fig). After acid treated soluble oligomerization, it was confirmed the presence of monomers and some oligomers in the reaction. The oligomers disappeared by subjecting the Nyl series enzymes and were converting into monomer units such as Ahx for Nylon-6, or HMD, and AD for Nylon-6,6, respectively. Additionally, 1H-NMR spectra of Nyl series treated samples matched the standards (S13, S14 Fig). These results demonstrate that the established chemo-enzymatic monomerization process can effectively convert high-molecular-weight nylon into monomers without any by-products.

I appreciate your keen and meaningful suggestions and comments.

Arigatou.

Reviewer #2: Shiraishi et al describe methods to convert nylon polymers into their monomeric component parts using a tandem chemical and enzymatic process. The manuscript focuses on two methodologies (1) converting nylons into microparticles using the well-understood solvent/non-solvent method, (2) chemically depolymerizing nylons using acids, with the resulting products subjected to enzymatic hydrolysis.

Whilst the recycling of nylons is a highly interesting topic, and the use of enzymes to recycle polymers has gained a lot of interest, the manuscript feels a little misleading in the way the methods used are being depicted. Firstly, the acid treatment is not a pre-treatment or limited, it is converting high molecular weight polymers to 5-mers, this is a significant amount of depolymerization! Secondly, the analytical methods chosen are not sufficient to support the claims made in the manuscript, as TLC based analysis cannot be used to identify "unknown" compounds alone, even if you suspect you know what those compounds are. Whilst the method presents an interesting route to the depolymerization of nylons, the use of enzymes does not seem warranted when it could be that optimizing the acid depolymerization step would be sufficient. The method is suggested to be a cost effective, environmentally friendly solution, however these things are not explicitly calculated or tested, so this cannot be claimed.

Major comments:

- Throughout the manuscript and in the title, the chemical step is described as a "chemical pre-treatment". This is simply not true; the described method is an acid catalyzed hydrolysis reaction which significantly deconstructs the polymer and is doing the bulk of the depolymerization. To call this a "pre-treatment" step is misleading and incorrect as the resulting mix of small oligomers can no longer be called a nylon polymer. In the PETase field grinding is considered a "pre-treatment" as it does not inherently change the chemical composition of the polymer or the polymer chain lengths, as this method does change both these things this sort of language needs to be revised. This should be updated throughout to describe what is actually being carried out i.e. extensive acid catalyzed depolymerization of the nylon followed by an enzymatic step. In addition, I really dislike the use of "limited hydrolysis" when describing the acid step. The depolymerization by the acid is actually very extensive.

Thank you very much for reviewer's insightful comments, which have helped improve our paper. We use the term "chemical pretreatment" to refer to chemical processes or reactions conducted in advance to facilitate the enzymatic reaction. As reviewer correctly pointed out, high-molecular-weight nylon is oligomerized by acid hydrolysis, which reduces its molecular weight. Therefore, we believe the use of this term is appropriate and does not pose any issues. We hope reviewer understand our usage of this term.

On the other hand, we agree that the term "limited hydrolysis" is not entirely accurate given the conclusions of this paper. Therefore, we replaced it with the term "soluble oligomerization," in the article as reviewer suggested.

- There is a misconception throughout that the enzymatic depolymerization of nylon is hampered by the effects of substrate crystallinity. This is incorrect. The depolymerization is affected by the strong intermolecular hydrogen bonding pattern between polymer chains. The acid treatment is not affecting the crystalline structure of the nylon at all, it is chemically depolymerizing it, turning the long polymer chains into shorter monomers which then do not have the same hydrogen bonding pattern. These short oligomers have no characteristic of "crystallinity" as they are no longer nylon polymer. This needs to be revised throughout the manuscript.

Thank you very much for reviewer's fruitful suggestions. We agree that it is too early to draw conclusions about the relationship between crystallinity and susceptibility to enzymatic hydrolysis. However, I believe reviewer will also agree that disrupting intermolecular hydrogen bonds is crucial for improving enzymatic degradation rates. With this in mind, I have revised the text as follows (Line 44-).

This complex nylon structure is determined by the intermolecular hydrogen bonding pattern between polymer chains. The enzymatic depolymerization is affected by its pattern because the strong intermolecular hydrogen bonding network makes nylon insoluble in water and also reduces the amount of exposed free amide bonds that the enzyme can attack.

- Again, for the crystallinity, as no DSC has been carried out on the polymer there is no evidence that the polymer used is highly crystalline. Crystallinity of nylons is also significantly affected by incubation in aqueous buffers, what is your evidence that the nylon in your reactions is significantly crystalline?

We thank the reviewer for their comments. As mentioned above, since the relationship between crystallinity and enzymatic hydrolysis efficiency is still unclear, we have removed any statements suggesting a direct correlation. Instead, we have revised the text to focus on the effect of hydrogen bond formation as above Q&A section.

- There are numerous grammatical errors throughout the introduction, and phrases which do not make sense like "low simple monomer units". The introduction needs to be carefully revised to remove these errors and inconsistencies.

We thank the reviewer for pointing out this word selection. To prevent any potential misunderstandings, we have implemented the following changes (Line 35-).

Nylon-6 and nylon-6,6 are composed of simple monomer units: nylon-6 consists of 6-aminohexanoate (Ahx), while nylon-6,6 is made from hexamethylenediamine (HMD) and adipic acid (AD).

- In the introduction, I don't think it is fair to say that acid hydrolysis increases the water solubility of

nylon, it is no longer really a nylon polymer by the time it is significantly hydrolyzed, what you are doing is turning it into more soluble oligomers of nylon.

Thank you very much for reviewer's insightful comments. We will use the term "soluble oligomerization," in the article as you suggested.

- For publication in PLOS ONE, the use of TLC as one of the major analysis methods is insufficient. TLC does not tell you what the products are and is not informative enough for the described study. There are far superior analysis methods which accurately detect and quantify nylon oligomers such as mass spectrometry, LC-MS, HPLC and LC-MS/MS. Phrases such as the monomer concentration was "estimated" to be zero are not appropriate when this could be rigorously analyzed.

We thank the reviewer for their comments. We have employed qualitative analysis via TLC and quantitative analysis using the TNBS method in our previous papers. We now, however, agree with your suggestion that it is important to confirm whether the product is indeed the desired Ahx, adipic acid, or hexamethylenediamine. Thus first, we showed the LC-MS analysis to confirm the formation of nylon oligomers after hydrochloric acid treatment, and demonstrate that they are broken down into monomeric components through the Nyl series treatment (S9-S12 Fig and added the sentences in main body at Line267-). Second, we used 1H-NMR analysis to confirm that the final product matches the authentic spectrum of Ahx for nylon-6, and hexamethylenediamine and adipic acid for nylon-66 (S13, 14 Fig and added the sentences in main body at Line270-).

Next, the molecular species present in the reaction mixture were tracked using LC-MS analysis (S9-S12 Fig). After acid treated soluble oligomerization, it was confirmed the presence of monomers and some oligomers in the reaction. The oligomers disappeared by subjecting the Nyl series enzymes and were converting into monomer units such as Ahx for Nylon-6, or HMD, and AD for Nylon-6,6, respectively. Additionally, 1H-NMR spectra of Nyl series treated samples matched the standards (S13, S14 Fig). These results demonstrate that the established chemo-enzymatic monomerization process can effectively convert high-molecular-weight nylon into monomers without any by-products.

- The TNBS method is insufficient as an analysis method for this study. It is not shown that the nylon oligomers of different lengths react with TNBS in the same manner, meaning values obtained by this method may be incorrect. The monomeric rate cannot be calculated from the TNBS method as it will also react with nylon oligomers of various sizes, especially in the case of NylC where the major product of the reaction is the dimer, not the monomer. This calculation is misleading and most likely incorrect, as all lengths of oligomer are being treated as "monomer".

We thank the reviewer for their comments. As the reviewer pointed out, the reaction system after acid treatment consists of a mixture of nylon oligomers of various chain lengths, as well as nylon monomers.

However, when treated with both NylB and NylC, the water-soluble oligomer components are converted to monomers, as confirmed through LC-MS, 1H-NMR, and TLC analysis. Based on these results, we do not believe that the monomerization rate indicated in this paper is incorrect.

- The use of nylon nano particle suspensions for enzymatic hydrolysis is already described (see https://onlinelibrary.wiley.com/doi/10.1002/anie.202404492), where water was used successfully as the non-solvent. Please could you comment on this discrepancy? This paper should be cited.

We sincerely thank the reviewer for their valuable comments. As pointed out, formic acid-based fine dispersion has already been reported in recent literature. We have now included this information and the relevant citation in the main text.

The reason formic acid can disperse efficiently, even when water is used as an antisolvent, may be due to the difference in water solubility between formic acid and TFE. We believe it is valuable for readers to see multiple methods proposed for dispersing nylon, as it provides a broader perspective on the available techniques. We have now included this information and the relevant citation in the main text (Line 152-).

Recently, a technique for the fine dispersions in aqueous solution of nylon-6 using formic acid has been developed, showing improved sensitivity to enzymatic hydrolysis.[24] Since formic acid has a concern to cleave amide bonds in polymeric nylon and reduce its molecular weight, we explored the use of TFE, another nylon-dissolving and low concern for cleaving amide bonds solvent, for dispersions in aqueous solution.

- The section on homogenous dispersion is very confusing. From line 149 onwards it is unclear what has been done. Were reactions carried out in solvent? (line 160).

We thank the reviewer for their comments. Apologies for the confusion. The dispersion method is detailed in Section 2. In this process, nylon is micronized by dropwise addition into an antisolvent, then washed with buffer to remove any residual solvent, and finally resuspended in an aqueous solution. This step is necessary because the Nyl series enzymes do not function effectively without it. Thank you for reviewer's understanding.

- Using the described analysis methods, the increase in release of monomers cannot be commented on (line 171) as described above.

We thank the reviewer for their comments. Apologies for the confusion. When the Nyl series is treated to nylon pellets, the monomerization rate is approximately 1%. However, when the Nyl series is used on nylon that has been homogeneous dispersed using ethylene glycol or ethanol as the antisolvent, the monomerization rate exceeds 10%. This is why the text states that over 10-fold greater enzymatic hydrolysis sensitivity is achieved.

- Line 177, what is the sedimentation? There is limited characterization of the acid treated polymer, so it is unclear what is in this reaction mixture, what size are the oligomers, what is the ratio of the different oligomer sizes?

We thank the reviewer for their comments. The sedimentation consists of nylon with a molecular weight that is insoluble in water. Although molecular weight measurements were not performed on all samples, Table 1 shows the results of molecular weight analysis after 168 hours of treatment in formic acid at 100°C. The supplemental section contains the GPC analysis results, which provide information on the molecular weight distribution and its ratio. We believe that the reader can assess the fragmentation status of pretreated nylon after each processing step from this information.

- You cannot identify molecules by TLC alone (line 192).

We thank the reviewer for their comments. Comparison with authentic samples of nylon-6,6 monomer units and their derivatives, synthesized in our laboratory, confirms that the observed spots correspond to the expected oligomeric components. As mentioned in the following later Q&A sections, LC-MS analysis further verified that each component aligns with these results. Therefore, although TLC is a qualitative method, we have used it to estimate the components present in the reaction system.

- The chemical catalyst is referred to as "more cost effective" but no cost analysis is done. Iridium is also a precious metal, will this scale well?

We thank the reviewer for their comments. We explored alternative methods for removing formic acid, considering that chemical catalysts are easier to handle than the lyophilization process. Ultimately, we did not perform a cost analysis of the catalyst because we achieved shorter reaction times using other acids, such as hydrochloric acid, compared to oligomerization with formic acid. However, the reaction efficiency of this catalyst is excellent, and studies are currently underway to explore its practical application.

- The conclusion claims "quantitative monomerization" but the analysis methods are insufficient to claim this.

We thank the reviewer for their comments. We have employed qualitative analysis via TLC and quantitative analysis using the TNBS method in our previous papers. We now, however, agree with your suggestion that it is important to confirm whether the product is indeed the desired Ahx, adipic acid, or hexamethylenediamine. Thus we useed the LC-MS and 1H-NMR analysis to confirm that the final product matches the authentic spectrum of Ahx for nylon-6, and hexamethylenediamine and adipic acid for nylon-66 as mentioned in above Q&A section.

- I think it is incorrect to claim this is ready for full-scale chemical recycling of nylon when there has been no TEA/LCA analysis. How would microparticles be created at scale?

We thank the reviewer for their comments. I believe your point is valid. However, at this stage of the research, the results are based on laboratory-scale experiments, and we did not pursue further verification because we do not consider it appropriate to conduct an LCA (Life Cycle Assessment) analysis at this point. It would be more suitable to perform an LCA analysis after scaling up to a more practical level, such as treating over 100 kg of PA. We have instead calculated the E factor as pointed out by reviewer 4 and added a description in the main body (Line 273-). We would appreciate reviewer's confirmation.

This paper also explores the appropriate chemical treatment for nylon recycling using enzymes, and in this process, we examined homogeneous dispersion methods. However, the enzymatic degradation efficiency of this method remains low, and it has not been optimized. As a result, we did not consider it necessary to verify the scale-up of the dispersion process. We appreciate your understanding in this matter.

Finally, to assess the environmental impact, the Environmental (E) Factor (kg waste / kg product) was calculated.[25] The values were 1.1 for nylon-6 and 1.3 for nylon-6,6, respectively. The ideal E Factor is zero, with higher values indicating more waste and, consequently, a greater negative environmental impact. However, we believe these calculated values are acceptable for chemical recycling methods for Nylons.

- The figure legends are not sufficient to replicate the results, what enzymes were used at what concentration? How were these enzymes expressed and purified?

We thank the reviewer for their comments. We added the information of enzyme preparation as following in experimental section (Line 84-), and also explained details in Supplemental section.

For these two enzymes, a His-tagged linker was fused to the N-terminal region. Details are described in Supplemental section.

- The images of the TLCs should be labelled with what the spots are estimated to be.

We thank the reviewer for their suggestions. We have already indicated the names of the authentic samples in the figure legend. To make identification easier at a glance, we have now added labels directly on the figure. Thank you for bringing this to our attention.

_ I could no find mention of how many replicates were conducted nor what the error bars on the graph represented.

We thank the reviewer for their suggestions. Each experiment was repeated at least three times under similar conditions (N = 3). The error bars represent the variability in the values obtained from each

experiment. This information has been added to the figure legend for clarity.

In conclusion, although this manuscript describes a potentially interesting approach, it requires significant revisions to be of sufficient caliber for publication in PLOS ONE. The analytical approach is quite poor and would need to be significantly revised and updated to support the claims being made, with proper analytics as described above. Furthermore, the misleading claims and language surrounding the acid depolymerization step needs to be removed, and the title updated to reflect this. There needs to be a significant effort to make the language and explanation of what was carried out clearer to avoid confusion. In addition, the figure legends and methods need to be updated so that all experimental details are included and could be carried out by others.

Thank you very much for your many helpful suggestions. We have revised the paper in accordance with the reviewer's comments to ensure that it conveys more accurate information to a broader audience. We truly appreciate your careful attention to the content and your valuable feedback. Thank you again for your support.

Arigatou.

Reviewer #3: Quantitative nylon monomerization by the combination of chemical pretreatment and enzymatic hydrolysis using nylon hydrolases

The study by Yuki Shiraishi and colleagues investigates the chemo-enzymatic depolymerization of polyamides 6 and 6,6. The authors compare different chemical pretreatments and the effects on physical properties of the polymers (Mw, Mn, particle size, etc.) and the subsequent enzymatic hydrolysis efficiency. Firstly, the authors investigate the effect of dispersing the nylon (in 6 different solvents) and of limited hydrolysis (using different acids, temperatures and reaction times), including the combination of the two chemical pretreatments, which allowed to increase the enzymatic degradation to 95% for both nylon types. Using stronger acids such as hydrochloric acids allowed to skip the dispersion step and shortened the pretreatment reaction time to only 2 h, compared to the 168h used with formic acid. Finally, the authors tested their process on used fishing nets, reaching 80% monomerization.

Currently, there is a very limited amount of studies dealing with the recycling of real EoL plastic waste, which makes this study of particular interest.

The manuscript is well written and easy to follow. The experimental set up is sound and the results are clearly presented.

The manuscript could be further improved by providing more advanced characterization of the depolymerization products (and the study of the mode of action of the enzyme on these oligomers), and explaining more in detail the enzyme part, so that other researchers could replicate the experiments. The total lack of explanation regarding the expression and purification of the enzyme is rather striking. I strongly recommend to either use some references or then explain in the material and methods the protocols used.

Some more detailed comments:

Lines 76-78: how were these enzymes obtained/produced for your experiments?

Are they expressed in E.coli? What protocol for expression and purification? What enzyme yields when operating at increasing nylon titers? Etc.

We thank the reviewer for their comments. We added the information of enzyme preparation as following in experimental section (Line 84-), and also explained details in Supplemental section. For these two enzymes, a His-tagged linker was fused to the N-terminal region. Details are described in Supplemental section.

-Lines 88-89: what about the hexamethylenediamine?

We thank the reviewer for their comment. The TNBS method detects amino groups and reflects their concentration, regardless of the molecular species present in the reaction system. Since we have confirmed that equivalent results can be obtained with both Ahx and hexamethylenediamine, we used

Ahx as the standard molecule for the calibration curve, even in the nylon-6,6 degradation experiments.

- line 96: please correct “EasIVial” into “EasiVial”

We thank the reviewer for pointing out a spelling error. Our mistake was corrected (Line 103).

- Line 108: 150 g/L nylon in water?

We thank the reviewer for their comment. Nylons do not dissolve or undergo any changes when heated in water. We have also confirmed that its sensitivity to enzymes remains completely unaffected.

- Line 212-123: please explain the rational why you used different times of incubations depending on the temperature (Section 3.1) and the type of acid used (2h, 96h, 168h)

We thank the reviewer for their comment. Initially, for the formic acid treatment, we set the reaction time to 96 hours (4 days) and compared the monomerization rates across three different heating temperatures. While we did not achieve satisfactory results at 75°C, we observed good results at 100°C and 120°C. Since we aimed to use the lowest possible processing temperature, we extended the heating time at 100°C to 168 hours (7 days) to see if this would improve the monomerization rate. However, we concluded that this prolonged reaction time was impractical for efficient chemical recycling, prompting us to explore other acids. In the case of hydrochloric acid, we found that an overnight reaction was sufficient for oligomerization, and after optimizing the conditions, we achieved a reaction time of just 2 hours. Through these studies, it became clear that the optimal reaction time varies significantly depending on the type of acid used.

- Line 133: please explain better how the enzyme solution was obtained

We thank the reviewer for their comment. The response to this comment is provided at the first Q&A section. We would greatly appreciate it if you could kindly check the information there.

- line 138: please elaborate better on how you determined the theoretical amino group concentration

We thank the reviewer for their comment. Nylon consists of molecules with varying degrees of polymerization, meaning its molecular structure is not uniform, and precise number of moles cannot be directly calculated. Therefore, we estimated the theoretical number of moles based on the molecular weight (Mw) of the monomer unit (i.e., Mw of the monomer molecule minus Mw of water molecule). This value was then used to estimate the maximum potential amount of amino groups present in the polymer. The monomerization rate was calculated by comparing the amount of amino groups quantified in the reaction system using the TNBS method against this theoretical value.

- line171: interesting that the larger particle size of nylon 6,6 did not negatively affect the enzymatic

depolymerization. This seems to differ from other studies and observations. You might elaborate a bit on this.

We thank the reviewer for their comments. We believe that the enzyme sensitivity of dispersed nylon in EG shows little difference between the two types, with degradation rates of 11% for nylon-6 and 14% for nylon-6,6. However, it is not yet clear whether this difference in degradation rates correlates with differences in the size of the nylon particles. One of our hypotheses is that the percentage of amorphous nylon on the particle surface may significantly affect the degradability by Nyl series enzymes, but we have not yet thought of a way to resolve this hypothesis.

- Line 192: how did you identify these monomer units exactly? Did you use specific standards?

We thank the reviewer for their comments. As reviewer mentioned, we use synthetic standard molecules to confirm the molecular species corresponding to the spots on the TLC.

- Line 195: as a comparison, the chemical pretreatments with dispersion and limited hydrolysis should be extended for an additional 36-72h (the time of enzymatic hydrolysis incubation) for a better comparison. This would allow to highlight better the contribution of the chemo-enzymatic compared to the chemical treatment alone.

We thank the reviewer for their comments. We have extended the enzyme incubation time to 72 hr, which is longer than the 24 hr described in our manuscript. However, the monomerization rate remained the same as with the 24 hr treatment, and no significant improvement was observed. Therefore, we believe the enzymatic reaction time (24 hr) is sufficient.

I appreciate your keen and meaningful suggestions and comments.
Arigatou.

Reviewer #4: Review of manuscript

PONE-D-24-29644

The authors present in their work entitled “Quantitative nylon monomerization by the combination of chemical pretreatment and enzymatic hydrolysis using nylon hydrolases” the combined chemical-biotechnological decomposition of two nylon derivatives, both in form of virgin pellets and one commercial nylon product for recycling purposes.

The work is well structured and written logically, and the description of the methods used for biomass fractionation and compound characterisation are sufficiently detailed such as to allow experts in the field to reproduce eventually. The data seem sound, and the conclusions drawn are justified by the various data presented. A few aspects should nevertheless be addressed during a revision:

• In the introduction, a small table might be interesting detailing the works done in the field with their characteristics, and highlighting the novelty of the current work. Some aspects are cited in the text, but an overview in table form might be more informative as such.

We thank the reviewer for their impressive suggestions. We have summarized as Table 1 that provides a summary of comparing our method with previously reported methods (Table 1 and Line 69-).

Table 1 provides a comparison between Shukla's methods and the chemo-enzymatic combination method proposed in this article.

• The number of significant figures used when describing the monomer yields might need to be reconsidered/checked.

We thank the reviewer for their comments. Since the molecular weight of polymers is not uniform and consists of a mixture of various molecular weights, we believe that comparing monomerization rates at the decimal level is not meaningful. Therefore, we kindly request that you accept the presentation of these values as whole numbers.

• The enzyme loading is relatively high. How easy can the products be isolated, and eventually purified/separated for a renewed use in a hypothetical circular economy setting?

We thank the reviewer for their comments. The primary goal of this paper is to establish the chemo-enzymatic reaction conditions for nylon monomerization. Therefore, at this stage, we have not conducted a study on the isolation and recovery of the products. However, there are numerous reports on the isolation of ω-amino acids, diamines, and dicarboxylic acids from aqueous solutions. For instance, the study by S.R.Shukla et al., which inspired this research, introduces a method for isolation using ion exchange resins. Other techniques, such as solvent precipitation, are also viable options. We believe that it is feasible to separate the monomer components from water and achieve repolymerization using these methods.

• How many times can the enzymes be technically reused, and how much activity do they lose during the process? How prone are they to be inhibited by impurities like colours, etc, that would be part of a nylon-based clothes, for example?

We thank the reviewer for their comments. We conducted a simple preliminary experiment on enzyme reuse and confirmed that the enzymes can be reused approximately five times by recovering them from the reaction system using ultrafiltration. This is likely due to the high thermal stability of the enzyme protein. We plan to develop a more detailed experimental system in the future to further investigate how many cycles of reuse the enzyme can withstand.

Another intriguing aspect is the potential for inhibition by additives. Given the wide variety of nylon composite materials available, we are actively investigating this issue, including which products to target for further study. We aim to publish a follow-up paper on this topic in the future.

However, the primary focus of this paper is to establish the chemo-enzymatic reaction conditions for nylon monomerization. We appreciate your understanding that we will not be addressing these topics in this paper.

• The main concern is connected to the real sample treated in this work. The paragraph and the information is rather limited, and some questions remain. For example, why was hydrochloric acid used in this case, and not formic acid? How easy is it to separate the hydrolysis products from the 20% remains? What do the TLCs look like for the real sample?

We thank the reviewer for their comments. Inspired by the work by S.R. Shukla et al., this study aims to achieve easier and complete monomerization through a combination of pretreatment using acid treatment and subsequent enzymatic hydrolysis. We selected these three acids, formic acid, hydrochloric acid, and sulfuric acid, because they used in their research on nylon oligomerization. Since the residue is primarily a precipitate, it can be easily separated from the hydrolysis products through filtration.

The results of TLC analysis are available in the Supplementary section (S15 Fig).

• Would it be possible to calculate the E-factor for the optimised process, i.e., prehydrolysis plus enzymatic depolymerisation?

Thank you very much for reviewer's fruitful suggestions. Following the methodology outlined by R.A. Sheldon, we calculated the E values for the optimized chemo-enzymatic process applied to nylon-6 and nylon-6,6. These values are described in the main text as follows (Line 273-).

Finally, to assess the environmental impact, the Environmental (E) Factor (kg waste / kg product) was calculated.[25] The values were 1.1 for nylon-6 and 1.3 for nylon-6,6, respectively. The ideal E Factor is zero, with higher values indicating more waste and, consequently, a greater negative

environmental impact. However, we believe these calculated values are acceptable for chemical recycling methods for Nylons.

I appreciate your keen and meaningful suggestions and comments.
Arigatou.
