# Supplementary material for: Quantitative nylon monomerization by the combination of chemical pretreatment and enzymatic hydrolysis using nylon hydrolases
Source: PLoS One. 2025 Feb 10;20(2):e0318641. doi: 10.1371/journal.pone.0318641 (PMC11809917; doi:10.1371/journal.pone.0318641)
Supplement: Attachment — Submitted filename: Response_to_Reviewers.pdf [file pone.0318641.s003.pdf]

To the editor and reviewers,

We would like to extend our sincere gratitude to all reviewers for their insightful and constructive comments. We have carefully revised our manuscript in accordance with the reviewers' suggestions. Thanks to their valuable feedback, we believe the revised manuscript now offers readers more accurate and detailed information compared to the original submission.

To address the revisions, it was necessary to perform additional Thermogravimetric analysis (TGA). As a result, we would like to propose adding the researcher, Sho Nonaka and Yoshiro Kaneko, who conducted this experiment as a co-author. We have obtained consent from the other co-authors, and we kindly ask for reviewers' consideration on this matter.

Below, we provide our responses to each comment, color-coded as follows: Reviewer comments are provided in black font. Our responses are shown in blue font. Changes to the manuscript are shown in red font.

Reviewer #2: The authors have made a good attempt to review the manuscript and have added additional data to support their claims. However, I have a number of suggestions that should be addressed prior to publication:

- In the response to the reviewer's comments, the authors raise some interesting hypotheses that are not referred to in the manuscript e.g. that amorphous nylon on the surface may affect deconstruction rates, enzyme inhibition by additives and enzyme reuse. These are all valuable points which should be mentioned in the conclusion to highlight where the field is going next. These points are important for understanding the context of the current manuscript.

We sincerely thank the reviewer for their valuable comments and insights.

- The new NMR and LC-MS data is far more compelling evidence of deconstruction than the original TLC images. I strongly suggest the NMR/LC-MS data is moved to a main figure in the manuscript and some of the TLC images can then be moved to the supplementary.

We thank the reviewer for their suggestions. According to their suggestions, LC-MS and 1H-NMR data were shown in the main text as Fig 7-12. We also added the following sentence in experimental section (Line 109-) and results and discussion (Line 292-), respectively.

1.8 Liquid chromatography-mass spectrometry (LC-MS) Analysis

LC-MS analysis was performed using an Alliance W2690/5 system (Nihon Waters K.K., Tokyo, Japan) equipped with a PDA Detector W2998 and an ACQUITY QDA Detector operating in positive ion mode. Separation was achieved on an XBridge C18 column (3.5 µm, 4.6 mm × 150 mm, Waters) maintained at 40 °C. The mobile phase consisted of Solvent A (10 mM ammonium acetate)

and Solvent B (acetonitrile). A gradient elution program was employed as follows: 0–10 min, 0–15% B; 10–20 min, 15–100% B; 20–30 min, 100% B; 30–40 min, 0% B. The flow rate was set at 0.5 mL/min.

1.9 1H-NMR measurement

The 1H-NMR measurements were conducted using a JNM-ECA600 nuclear magnetic resonance spectrometer (JEOL JNM-ECA600KS, JEOL DATUM LTD., Tokyo, Japan). The sample was ultrafiltered to remove the Nyl series proteins, lyophilized, and subsequently dissolved in deuterium oxide (D_2O) to prepare for analysis.

Additionally, the 1H-NMR spectra of Nyl series treated samples matched the standards and showed only peaks corresponding to the respective monomer units and glycerol, which was included in BufferA (Fig 11,12).

- Even with the calculated E factor, I still think it is too early to suggest this is a scalable, economically feasible and environmentally feasible solution to nylon waste. It would be more honest for the authors to mention in the conclusion that this is an exciting new avenue to explore, but TEA and LCA will need to be used down the line to confirm that it is economically feasible method, and that scaling up will also be important to test in the future.

We thank the reviewer for their comment. We added the following sentence in results and discussion (Line 310-) and in conclusion section (Line 332-), respectively.

While a more detailed investigation into the applicability of this value will be necessary following scale-up studies, the calculated value is thought to be acceptable for a chemical recycling of nylon on a laboratory scale.

While there are still many factors to consider in the future, such as techno-economic analysis (TEA), life cycle assessment (LCA), and scale-up studies to confirm the economic feasibility of the method, this study has demonstrated the effectiveness of our approach on market products such as used fishing nets. We believe this underscores the significant potential of our method for achieving full-scale chemical recycling of nylon products in near future.

- I still believe a DSC for the nylons pre- and post- treatment really should be included to help other researchers replicate the research.

We thank the reviewer for their suggestions. Initially, we planned to perform DSC measurements; however, due to the risk of sample volatilization potentially damaging the equipment, we opted for TGA measurements to evaluate the differences before and after acid treatment. The TGA results revealed entirely distinct behavior compared to the starting nylon pellets. By combining these findings

with subsequent LC-MS and 1H-NMR analyses, we aim to clearly demonstrate to readers the changes in the samples induced by the pretreatment process. Specifically, thermogravimetric analysis (TGA) data were shown in supporting data as S9, S10 Figs. We also added the following sentence in experimental section (Line 106-) and results and discussion (Line 283-), respectively.

1.7 Thermogravimetric analysis (TGA)

TGA was performed using TGA-50 (SHIMADZU Co., Kyoto, Japan). The samples were heated from room temperature (ca. 25°C) to 1,000°C at a heating rate of 10°C/min under nitrogen flow (100 mL/min).

The samples, both before and after acid and/or enzyme treatments, were analyzed using TGA, LC-MS, and 1H-NMR techniques. S9 and S10 Fig. present the TGA data for nylon pellets and the acid caused solubilized samples. The results reveal distinct differences in thermal decomposition behavior between the original nylon pellets and the acid caused solubilized samples. While the nylon pellets decomposed around 400°C, the acid caused solubilized samples exhibited a three-step decomposition, with the first stage beginning at approximately 200°C. This initial degradation aligns with the behavior observed for nylon monomers such as Ahx and AD-HMD salt. The second stage of degradation occurred at around 400°C, consistent with the thermal behavior of high molecular weight nylons. The constant residue observed between 500°C and 800°C was attributed to NaCl, generated during the neutralization process. These findings suggest that the acid caused solubilized samples contain significant amounts of monomeric and oligomeric components.

- There are still instances of some confusing sentence structures: e.g.“formic acid has a concern to cleave amide bonds”. I strongly suggest the manuscript is carefully checked prior to publication to aid reading clarity.

We thank the reviewer for their suggestion. We inserted and changed around sentences as following in results and discussion (Line 166-).

Recently, a technique for achieving fine dispersions of nylon-6 in aqueous solutions using formic acid has been developed, demonstrating enhanced sensitivity to enzymatic hydrolysis.[24] However, formic acid raises concerns about cleaving amide bonds in polymeric nylon, leading to a reduction in molecular weight. We explored the use of TFE, an alternative nylon-dissolving solvent with minimal risk of amide bond cleavage, for creating aqueous dispersions.

If the above comments are taken into consideration, I am happy to recommend the manuscript for publication.

I appreciate your keen and meaningful suggestions and comments.

Arigatou.

Reviewer #4: The authors present the revised version of their work entitled “Quantitative nylon monomerization by the combination of chemical pretreatment and enzymatic hydrolysis using nylon hydrolases”.

The authors revised the manuscript carefully, and responded in a satisfying manner to the reviewers’ comments. The paper seems suitable for publishing.

As a last minor aspect, the authors should check the wording when using the technical term soluble oligomerisation. For example, rather than writing ‘formic acid treated soluble oligomerization’, it might be better to formulate: ‘formic acid caused soluble oligomerisation’, ‘soluble oligomerization upon formic acid treatment’ or ‘soluble oligomerisation induced by formic acid treatment’. Currently one could get the impression that the term has just been added to the sentence.

Thank you very much for the reviewer’s insightful comments, which have greatly contributed to improving our paper. We have revised the term "acid treated soluble oligomerization" to the suggested "acid caused soluble oligomerization" in our manuscript.

I appreciate your keen and meaningful suggestions and comments.

Arigatou.
